# Higher ultra-processed food intake is associated with an increased incidence risk of cardiovascular disease: the Tehran lipid and glucose study

**DOI:** 10.1186/s12986-024-00788-x

**Published:** 2024-03-19

**Authors:** Mohammad Jalali, Zahra Bahadoran, Parvin Mirmiran, Davood Khalili, Michael E. Symonds, Fereidoun Azizi, Shiva Faghih

**Affiliations:** 1 Department of Community Nutrition, School of Nutrition and Food Sciences, Shiraz University of Medical Sciences, Razi Ave, Shiraz, Iran; 2 Nutrition and Endocrine Research Center, Research Institute for Endocrine Sciences, Shahid Beheshti University of Medical Sciences, Shahid-Erabi St., Yeman St., Velenjak, Tehran, Iran; 3 Department of Clinical Nutrition and Dietetics, Faculty of Nutrition and Food Technology, National Nutrition and Food Technology Research Institute, Shahid Beheshti University of Medical Sciences, No. 24, Shahid-Erabi St., Yeman St., Velenjak, Tehran, Iran; 4 Department of Epidemiology and Biostatistics, Research Institute for Endocrine Sciences, Shahid Beheshti University of Medical Sciences, Tehran, Iran; 5 Prevention of Metabolic Disorders Research Center, Research Institute for Endocrine Sciences, Shahid Beheshti University of Medical Sciences, Tehran, Iran; 6 Centre for Perinatal Research, Academic Unit of Population and Lifespan Sciences, School of Medicine, University of Nottingham, Nottingham, NG7 2UH UK; 7 Endocrine Research Center, Research Institute for Endocrine Sciences, Shahid Beheshti University of Medical Sciences, Tehran, Iran

**Keywords:** Ultra-processed food, Cardiovascular disease, Salty snacks, Nutrition, Prospective observational study

## Abstract

**Background:**

Cardiovascular disease (CVD) is a major cause of death worldwide, although limited data are currently available regarding the impact of consuming ultra-processed food (UPF) on its incidence. Given the increased consumption of UPF in Iran, we aimed to investigate the association between UPF intake and CVD risk.

**Methods:**

Individuals without CVD (n = 2050) aged ≥ 30 years old were recruited from the Tehran Lipid and Glucose Study (TLGS). Dietary data were collected using a validated food frequency questionnaire (FFQ) and UPF intakes were assessed based on the Nova food classification. Multivariable Cox proportional hazard models adjusted for potential confounders were used to estimate the hazard ratio (HR) and 95% confidence intervals (95% CI) for the risk of CVD across tertiles of UPF intake.

**Results:**

A 10.1% incidence of CVD occurred over a median follow-up of 10.6 years, with a 22% increase in CVD risk per each 50 g/day UPF intake. Participants with the highest intake of UPF had a 68% greater incidence of CVD compared to those with the lowest intake (HR = 1.68, 95% CI=1.14–2.48) after controlling for potential confounders. Regarding sub-groups of UPF, participants in the 3rd tertile compared to the reference had a significantly increased risk of CVD (HR = 1.56, 95% CI=1.04–2.34). Nevertheless, intake of bread, fast food, sweetened beverages, sweets and desserts, high-fat dairy products, and other UPFs were not associated with greater CVD risk.

**Conclusion:**

Our findings support the hypothesis that the incidence of CVD is enhanced with the higher consumption of UPF in a representative sample of the Iranian population.

**Supplementary Information:**

The online version contains supplementary material available at 10.1186/s12986-024-00788-x.

## Introduction

Cardiovascular disease (CVD) remains the leading cause of death worldwide, with an estimated 18.6 million deaths in 2019, of which 58% occurred in Asia [1, 2]. Diet is a major factor contributing to CVD, causing nearly 70% of annual deaths [3], and is involved in the progression of atherosclerotic plaques, hypertension, and obesity [4]. In particular ultra-processed food (UPF), whose consumption has globally increased over the past two decades, is unhealthy because it is rich in salts, added sugar, saturated and trans fats, and poor in fiber, minerals, and vitamins [5–7]. The consumption of UPF accounts for 20–60% of daily energy intake in different populations [8]. Moreover, food processing modifies its chemical, physical, and nutritional composition with the potential to limit the bioavailability of nutrients in the small intestine [9, 10] and thus Cardiometabolic health [11].

Several studies have assessed the role of UPF on CVD risk factors such as hypertension [12], atherosclerosis [13], obesity [10], dyslipidemia [14] and type 2 diabetes [15, 16]. Few studies, however, have investigated the association between UPF consumption and the risk of CVD [9, 17]. Furthermore, current data are restricted to populations in the USA and Europe. The current population-based cohort study was therefore designed within the framework of the Tehran Lipid and Glucose Study (TLGS) to investigate the association between UPF intake and CVD risk in Iranian adults.

## Materials and methods

### Study design and population

TLGS is an ongoing large-scale community-based prospective cohort study carried out in the capital of Iran, to monitor the prevalence and incidence of non-communicable diseases (NCD) and establish healthy lifestyles to reduce risk [18]. The study protocol is based on the WHO-MONICA protocol for population surveys [19]. The study population was selected through a multistage stratified cluster random sampling technique from the population of district 13 in Tehran. District 13 was chosen mainly because city-wide data showed a high rate of stability in that district. The distribution and prevalence of cardiovascular risk factors in district 13 were representative of the overall population in Tehran [20]. In order to update the data on demographics, lifestyle, biochemical and clinical information, and anthropometric examination, participants were followed up every 3 years; the baseline survey was a cross-sectional study conducted from 1999 to 2001, and phases 2 (2002–2005), 3 (2006–2008), 4 (2009–2011), 5 (2012–2015), and 6 (2016–2019) were prospective follow-up surveys.

All of the TLGS participants signed an informed written consent document. The ethics committee of the Research Institute for Endocrine Sciences, Shahid Beheshti University of Medical Sciences, Tehran, Iran, approved the study protocol, which was conducted based on the Declaration of Helsinki (IR.SUMS.SCHEANUT.REC.1401.031).

As the nutritional data were added to the TLGS protocol from the third phase, the current longitudinal analysis was conducted on data from 2006 to 2008 to the end of March 2018. The analytical sample included 2050 adults aged ≥ 30 years, free from CVD at baseline, with completed dietary data and key variables i.e. demographics, clinical and biochemical profiles.

### Data collection and measurements

At the primary stage, participants were requested to complete a set of questionnaires about a wide array of characteristics related to sociodemographics and lifestyle (e.g. sex, date of birth, smoking status, anthropometry measures, dietary intakes, physical activity), and health status (personal and family history of diseases, medical treatments). To minimize the rate of missing data, all recall interviews were performed at participants’ homes. Trained interviewers double-checked and resolved any doubts on records.

Trained dietitians with at least 5 years experience in the TLGS survey assessed dietary intakes using the Iranian-validated version of the 168-items semi-quantitative Food Frequency Questionnaire (FFQ) [21, 22], which was designed to assess the frequency and portion size of food, on a daily, weekly, monthly or yearly basis. A standard portion size was designated for each food using the United States Department of Agriculture (USDA) serving size. To calculate daily energy, food, and nutrient intake, we converted the participants' responses to daily values and multiplied these by the USDA Food Composition Table (FCT) [23]. In the case of local foods absent from the USDA FCT, Iranian FCT was used as an alternative for traditional Iranian food items [24].

We assessed participants’ body weight while they were minimally clothed, without footwear, using a digital scale with an accuracy of 100 g (Seca, Hamburg, Germany). In addition, a calibrated stadiometer was used to measure the participant’s height to the nearest 0.1 cm standing in normal alignment. Body mass index (BMI) was then calculated by dividing weight (kg) by height squared (m^2^). A tape meter was used to record waist circumference (WC) at the end of normal expiration, over light clothing to the nearest 0.1 cm. The Iranian version of the Modifiable Activity Questionnaire (MAQ) [25] was undertaken to assess physical activity level, which was expressed as metabolic equivalent hours per week (MET-h/week.).

Before measuring blood pressure, participants were asked to have at least 15 min rest. Measurment was then undertaken by a qualified physician using a standard mercury sphygmomanometer calibrated by the Iranian Institute of Standards and Industrial Research [26]. Extended details regarding blood pressure measurement in the TLGS could be found elsewhere [27].

Blood samples were collected between 07.00 and 09.00 h from participants after 12–14 h of overnight fasting. These were centrifuged within 30–45 min of collection and the Pars Azmoon kits (Pars Azmoon Inc., Tehran, Iran) and Selectra 2 auto-analyzer (Vital Scientific, Spankeren, The Netherlands) were used to analyze all samples at the TLGS research laboratory. Fasting serum glucose (FSG), serum triglycerides (TG), and total cholesterol were measured by the enzymatic colorimetric method, using glucose oxidase, glycerol phosphate oxidase, and cholesteryl ester hydrolase, respectively. Serum high-density lipoprotein cholesterol (HDL-C) was measured by the HDLC Immuno FS kit. Both inter- and intra-assay coefficients of variations (CVs) were ≤ 2.9% [28].

### Exposure variable: ultra-processed foods

**Fig. 1:**
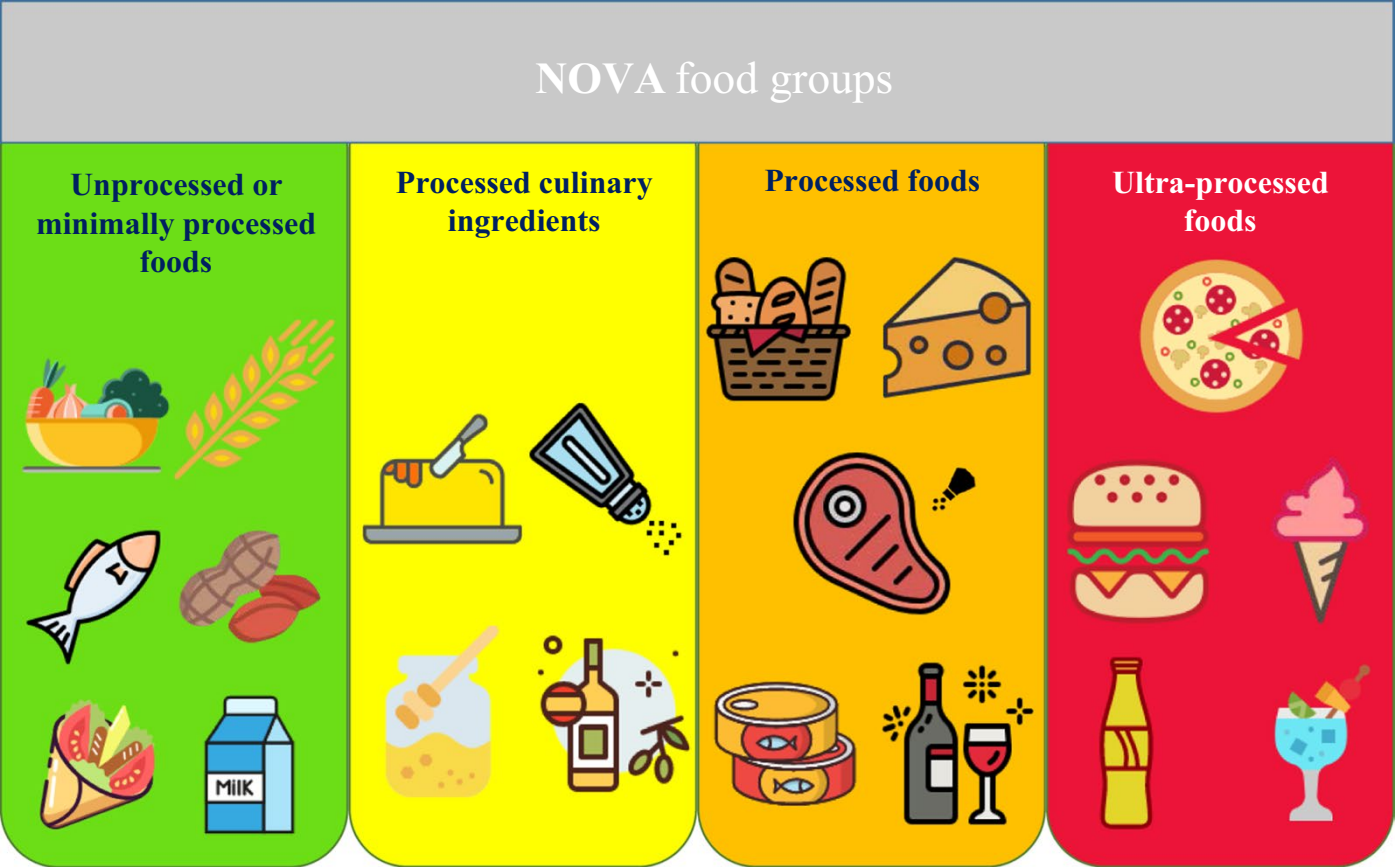
NOVA food classification and some examples for each groups

UPFs were selected using the Nova food classification, which categorizes foods based on the purpose, nature, and degree of food processing [29], and classified them into four groups (Fig. 1): (1) "Unprocessed or minimally processed foods", including fresh, dried or frozen plant and animal foods. (2) "Processed culinary ingredients", including sugar, oils, fats, salts, and other ingredients used in kitchens to prepare food. (3) "Processed foods", including canned fish and vegetables, plain bread, and homemade cheeses. And (4) "Ultra-processed foods": such as packaged or industrial breads, sweet or savory packaged snacks, industrial sweets and desserts, carbonated and sweetened beverages, and other food products made mostly or entirely from sugar, oils and fats, and other substances not commonly used in culinary preparations such as hydrogenated oils, modified starches, and protein isolates. Further definitions and details regarding Nova food groups are described in Additional file 1: Table S1 [30]. The list of FFQ items and selected UPF can be found in Additional file 2: Table S2. If there were any disagreements in the selection of UPF, a consensus was achieved through a group discussion.

### Definitions of terms and outcomes

Details of the ascertainment of CVD outcome have been published previously [31, 32], where CVD was defined as any coronary heart disease (CHD) event, stroke (a new neurological deficit that lasted ≥ 24 h), CVD death (definite fatal myocardial infarction (MI)), definite fatal stroke and definite fatal CHD. CHD events included cases of definite MI (diagnostic electrocardiographic (ECG) results and biomarkers), probable MI (positive ECG findings plus cardiac symptoms or signs plus missing biomarkers or positive ECG findings plus equivocal biomarkers), angiographic proven CHD, unstable angina pectoris (new cardiac symptoms or changing symptom patterns and positive ECG findings with normal biomarkers) and CHD death. Previous ischemic heart disease and/or cerebrovascular events were defined by a history of CVD [31]. All diagnoses were made according to the 10th edition of the International Classification of Diseases codes (ICD-10) and American Heart Association (AHA) classification for cardiovascular events.

### Covariates

Hypertension (HTN) was defined as a systolic blood pressure (SBP) ≥ 140 mmHg, or diastolic blood pressure (DBP) ≥ 90 mmHg, or self-report of taking blood pressure medications [33]. Type 2 diabetes for participants was defined as those who met at least one of the following criteria: fasting serum glucose ≥ 126 mg/dL, or 2 h blood glucose ≥ 200 mg/dL, or being on anti-diabetic medication [34]. The CVD risk score was measured according to the sex-specific “general CVD” algorithm including age, smoking, SBP, treatment for HTN, total cholesterol, HDL-C, type 2 diabetes (T2D), and use of related medications [35]. This score has been validated among Iranians and is the main predictor of CVD events in the TLGS population [36].

### Statistical analysis

Mean, and standard deviation (SD) for continuous variables or the frequency (%) of baseline characteristics for categorical values were compared between tertiles of UPF intake (Table 1) using the analysis of variance (ANOVA), and between CVD and non-CVD cases (Additional file 4: Table S4) applying an independent sample t-test or χ^2^ tests when appropriate. Given the non-normal distribution, the Mann–Whitney U test was performed. To report the amount of daily energy intake and dietary components (protein, carbohydrates, fats, cholesterols, saturated fats, and fibers) across tertiles of UPF intake and related correlations, we used ANOVA.

We applied the Cox proportional hazards model to obtain hazard ratios (HRs) and 95% confidence intervals (CIs) of CVD incidents across intake tertiles of UPF along with continuous variables (each 50 g/day). Time to event for CVD was defined as time to end of follow-up (censored cases) or time to having an event, whichever occurred first. We censored participants at the time of death due to non-CVD causes, at the time of leaving the district, or end of follow-up (March 2018).

The mean value of UPF intake (g/day) within the third to sixth phases was estimated as the main exposure variable to be included in the analysis. In addition to investigating the potential association between UPF and CVD risk, we conducted UPF subgroup analysis to assess if there is a relationship between specific UPF group (g/week) and CVD risk. The food items of each specific UPF subgroup are shown in Additional file 3: Table S3. In addition to crude, three other Cox models were conducted, model 1 was adjusted for the CVD risk score, which allowed us to include known CVD confounders without adding many variables, and improving the model stability; model 2 was additionally controlled for physical activity (hours/week); model 3 (full model) was additionally adjusted for energy intake (kcal/day) and dietary fiber (g/day).

Data analyses were operated using the Statistical Package for Social Sciences (version 22; SPSS). A *P*-value of < 0.05 represented statistical significance.

## Results

### Baseline details

At baseline, participants were middle-aged (mean (SD) age: 46.28 (11.34)), and 46% were male. In comparison with the 3rd and reference tertile, the smoking rate was higher in the group who consumed more UPF (*P* < 0.001). In contrast, SBP, serum cholesterol, diabetes rate (%), and FBS were lower in participants who consumed less UPF compared with the 3rd tertile (Table 1). During a median (IQR) of 10.6 (9.9–11.1) years of follow-up, 208 participants (10.1%) developed CVD. Participants who developed CVD tended to be older, more likely to be male, with a higher WC, SBP, DBP, FBS, serum cholesterol, TG to HDL ratio, and incidence of HTN, and diabetes in comparison with participants without CVD (*P* = 0.001) (Additional file 4: Table S4). On the other hand, differences in BMI, current smoking status, and physical activity were not statistically significant between CVD cases and non-cases (Additional file 4: Table S4).

**Table 1:** Baseline characteristics of the study participants (n = 2050)

Baseline characteristics	Total (n = 2050)	Tertile of UPFs			*P*
		Q1 (n = 683)	Q2 (n = 684)	Q3 (n = 683)	
Age (year)	46.28 (11.34)	51.65 (11.46)	44.90 (10.69)	42.29 (9.67)	< 0.001
Male (%)^#^	46.0	33.8	45.2	59.0	< 0.001
BMI	28.08 (4.62)	28.22 (4.64)	27.90 (4.59)	28.12 (4.64)	0.42
WC (cm)	92.99 (11.98)	92.98 (11.73)	92.24 (11.94)	93.74 (12.22)	0.07
HTN (%)^#^	16.9	24.5	14.3	11.9	< 0.001
SBP	114.77 (17.72)	118.61 (19.83)	113.55 (17.05)	112.15 (15.35)	< 0.001
DBP	75.27 (10.81)	75.71 (11.24)	75.24 (10.56)	74.86 (10.62)	0.35
Serum cholesterol	195.39 (37.90)	198.79 (39.51)	194.88 (38.57)	192.51 (35.27)	0.008
Diabetes (%)^#^	9.2	17.6	5.6	4.4	< 0.001
Fasting serum glucose (mg/dL)	94.97 (25.95)	100.56 (32.93)	92.40 (19.78)	91.97 (22.38)	< 0.001
Current smoking (%)^#^	13.7	8.1	13.0	20.1	< 0.001
Physical activity* (MET-hours/week)	15.8 (4.2–39.6)	17.0 (5.5–36.7)	15.8 (3.9–38.2)	15.8 (3.7–47.0)	0.06
TG to HDL ratio	4.23 (3.30)	4.18 (3.14)	4.20 (3.59)	4.30 (3.15)	0.79

Data are mean ± SD unless stated otherwise

*CVD* cardiovascular disease, *BMI* body mass index, *WC* waist circumference, *HTN* hypertension, *SBP* systolic blood pressure, *DBP* diastolic blood pressure, *FSG* fasting serum glucose, *TG* triglyceride, *HDL* high density lipoprotein

*Data is median (IQR); ^#^data are shown in percent (%)

Baseline dietary intakes are summarized in Table 2. In compared with participants in the lowest tertile of UPF intake; those in the third tertile had a higher dietary intake of daily energy, proteins, carbohydrates, total fats, cholesterols, saturated fats and fibers.

**Table 2:** Baseline dietary intakes of the study participants across tertiles of ultra-processed food consumption

Dietary intakes	Total (n = 2050)	Tertiles of UPF		
		T1 (n = 683) 27.10–59.29 (39.16 g/d)	T2 (n = 684) 59.36–105.01 (80.97 g/d)	T3 (n = 683) 105.03–175.30 (145.63 g/d)
Ultra-processed food subgroups				
Breads (g/week)	22.86 (7.49–68.6)	10.89 (0.00–23.49)	24.10 (11.73–68.6)	46.68 (22.86–100)
Salty snacks (g/week)	10.21 (1.02–34.86)	1.96 (0.00–8.16)	11.69 (2.10–33.83)	33.97 (11.76–87.5)
Fast foods (g/week)	72.4 (34.72–136)	34.12 (14.00–64.6)	77.1 (45.07–134)	124 (73.9–191)
Sweetened beverages (mL/week)	65.3 (10.71–228)	10.73 (0.00–65.3)	65.3 (21.43–137)	280 (65.3–560)
Sweets and desserts (g/week)	119 (60.8–211)	63.1 (29.89–107)	125.7 (70.5–199)	204 (126–287)
Dairy products (g/week)	65.2 (19.81–147)	19.83 (6.51–53.4)	75.0 (28.00–130)	147 (60.0–290)
Other UPF (g/week)	19.20 (7.00–48.00)	7.70 (2.58–19.20)	21.49 (7.70–47.98)	34.20 (17.73–71.7)
Total energy intake (kcal/day)	2309 ± 796	1987 ± 664	2264 ± 720	2675 ± 839
Total protein intake (% energy)	13.67 ± 2.17	14.19 ± 2.29	13.67 ± 2.16	13.14 ± 1.9
Total carbohydrate intake (% energy)	57.77 ± 6.67	59.36 ± 6.68	57.67 ± 6.62	56.29 ± 635
Total fat intake (% energy)	30.72 ± 6.02	30.69 ± 6.27	30.80 ± 6.19	30.66 ± 5.58
Total cholesterol intake (g/1000 kcal)	93.67 ± 31.12	89.81 ± 30.58	95.71 ± 32.99	95.47 ± 29.38
Saturated fat intake (g/1000 kcal)	11.25 ± 2.77	11.19 ± 3.07	11.28 ± 2.71	11.29 ± 2.49
Total fiber intake (g/1000 kcal)	16.67 ± 5.49	17.91 ± 5.80	16.57 ± 5.46	15.53 ± 4.93

Data are median (Interquartile range) or mean ± SD

### Ultra-processed foods intake and cardiovascular disease

Adjusting for CVD-risk score (model 1) resulted in an increased risk of CVD in the group with highest consumption of UPF in comparison with lowest one (HR = 1.62, 95% CI=1.16–2.26). The association was potentiated following further adjustment for physical activity (model 2) (HR = 1.69, 95% CI=1.19–2.41). Additional adjustment for energy and fiber intake in the fully-adjusted model showed that participants with highest intake of UPF had a 68% increased risk of CVD incidence compared to those who had lowest intake (145.63 vs. 39.16; HR = 1.68, 95% CI=1.14–2.48). Furthermore, continuous variable analysis showed a 22% increased risk of CVD events associated with each 50 g/day increased intake of UPF (HR = 1.22, 95% CI=1.03–1.45) (Table 3).

**Table 3:** Associations between Ultra-Processed Food Intake and CVD Incidents

Cox models	Continuous^$^	Tertiles of UPF intake		
		T1 (n = 683)	T2 (n = 684)	T3 (n = 683)
Crude	1.22 (1.05–1.41)*	1.00 (ref.)	1.07 (0.76–1.49)	1.65 (1.18–2.31)**
Model1	1.20 (1.04–1.40)*	1.00 (ref.)	1.06 (0.76–1.49)	1.62 (1.16–2.26)**
Model2	1.23 (1.05–1.44)**	1.00 (ref.)	1.13 (0.79–1.61)	1.69 (1.19–2.41)**
Model3	1.22 (1.03–1.45)*	1.00 (ref.)	1.13 (0.79–1.63)	1.68 (1.14–2.48)**

Cox regression models were used. Model 1: Adjusted for CVD-risk score; Model 2: Additionally adjusted for physical activity; Model 3: additionally adjusted for total energy intakes (kcal/d), and dietary intakes of fiber (g/d)

^$^Intakes of UPF based on each 50 g/day

*Statistical significance at *p* < 0.05, **statistical significance at *p* < 0.01

Regarding sub-groups of UPF, participants who consumed higher salty snacks had a significantly increased incidence of CVD (HR = 1.56, 95% CI=1.04–2.34). Nevertheless, the intake of bread, fast foods, sweetened beverages, sweets and desserts, high-fat dairy, and other UPF were not statistically associated with CVD risk (Table 4).

**Table 4:** Association between intakes of specific ultra-processed foods (g/week) and CVD incidents

UPF groups	Tertiles of sub-groups intake		
	T1 (n = 683)	T2 (n = 684)	T3 (n = 683)
*Breads*			
CVD Score-adjusted model^#^	1.00 (ref.)	1.32 (0.95–1.82)	1.29 (0.90–1.85)
Multi-variable adjusted model^$^	1.00 (ref.)	1.29 (0.92–1.82)	1.22 (0.83–1.80)
*Salty snacks*			
CVD Score-adjusted model	1.00 (ref.)	1.20 (0.87–1.65)	1.53 (1.04–2.25)*
Multi-variable adjusted model	1.00 (ref.)	1.30 (0.92–1.83)	1.56 (1.04–2.34)*
*Fast foods*			
CVD Score-adjusted model	1.00 (ref.)	1.33 (0.97–1.84)	1.28 (0.88–1.85)
Multi-variable adjusted model	1.00 (ref.)	1.41 (0.99–1.99)	1.32 (0.90–1.94)
*Sweetened beverages*			
CVD Score-adjusted model	1.00 (ref.)	1.17 (0.85–1.62)	1.35 (0.96–1.91)
Multi-variable adjusted model	1.00 (ref.)	1.20 (0.85–1.69)	1.32 (0.91–1.91)
*Sweets and desserts*			
CVD Score-adjusted model	1.00 (ref.)	1.17 (0.85–1.59)	0.98 (0.68–1.43)
Multi-variable adjusted model	1.00 (ref.)	1.14 (0.82–1.59)	0.94 (0.63–1.40)
*High-fat dairy*			
CVD Score-adjusted model	1.00 (ref.)	1.10 (0.80–1.51)	1.01 (0.69–1.47)
Multi-variable adjusted model	1.00 (ref.)	1.01 (0.72–1.43)	1.05 (0.70–1.56)
*Other*			
CVD Score-adjusted model	1.00 (ref.)	0.89 (0.65–1.23)	1.20 (0.84–1.71)
Multi-variable adjusted model	1.00 (ref.)	0.91 (0.65–1.27)	1.24 (0.84–1.81)

The list of food items considered for each subgroups could be find in Additional file 3: Table S3

^#^Model: adjusted for CVD score

^$^Model: additionally adjusted for PA, Calorie intake and dietary fiber

*Statistical significance at *p* < 0.05

## Discussion

The present prospective study with a median follow-up duration of 10.6 years examined the association between UPF consumption and CVD in Iranian adults. A daily increase of 50 g of UPF was linked with a 22% increased risk of CVD, which was independent of known confounding factors. Additionally, higher consumption of salty snacks was directly associated with CVD events.

Across the globe, there is a growing trend of relying on ultra-processed foods, which are ready-to-eat or heat products made with numerous additives and lack whole foods [30, 37]. UPF groups are widely available in middle- and low-income countries; and have replaced traditional and freshly prepared meals [38]. These foods have been identified as a significant contributor to the increase in chronic diseases related to diet such as diabetes, cancer and obesity [39–41]. Additionally, national studies have revealed that diets high in ultra-processed foods are nutritionally unbalanced. Therefore, processing level is now recognized as an essential aspect of diet quality that considers the qualitative nature of food [42], and some epidemiological studies have revealed that higher intake of UPF is associated with lower nutritional quality [43].

Whereas direct association between UPF intake and CVD events in adults has been reported in the NutriNet-Santé [9], and prospective Framingham Offspring Cohort studies [17]; results from the US National Health and Nutrition Examination Study (NHANES) [44] and SUN prospective cohort have failed to prove it [45]. In addition to different follow-up durations, the inconsistent results might be related to the potential differences in sociodemographic traits e.g. age, income, education, weight status, and time scarcity, which have been reported to be associated with UPF intake [46, 47].

Several biological mechanisms could explain the impact of UPF on CVD risk. They can increase the intake of sodium [48, 49], free sugar and *trans*-fats [50]; and modify glycemic responses [51], thereby promoting weight gain, inflammation, oxidative stress, and endothelial dysfunction following hyperglycemia [52]. UPF are also calorie-dense and less satiating thereby facilitating excessive energy intake [50] and thus increasing CVD risk [53].

In addition to nutritional aspects, newly formed compounds generated during food processing could lead to an increased risk of CVD. These include acrolein, a toxic compound generated during frying or cooking by heating the fats or oils and found in UPF, which could increase the  risk of CVD by causing vascular damage [54] and systemic dyslipidemia [55]. Dry-heat processing in addition to deep-frying in the preparation of ultra-processed salty snacks such as french fries and chips promotes the formation of advanced glycation end-products (AGEs) that can contribute to CVD [56]. Dietary emulsifiers added to food in the industrial process appear to be linked with metabolic syndrome and chronic inflammation by disrupting gut microbiota integrity, and augmenting its pro-inflammatory potential by increasing microbiotic virulence factors [57, 58]. A newly raised concern regarding industrial packaging materials for UPF refers to the potential adverse effect of bisphenol A (BPA) on CVD and its major risk factors including insulin resistance, hypertension, abdominal obesity, atherosclerosis, and oxidative stress [59]. Increased use of inorganic phosphate salts as an additive to the UPF could contribute to the development and progression of CVD by promoting endothelial dysfunction and vascular calcification by inhibitory effects on the renal activation of 25-hydroxyvitamin D to the active metabolite 1,25D [60].

### Strengths and limitations

The prospective design, with a long-term follow-up and relatively large sample size, could be considered as a major strength of our study, with CVD outcomes based on medical records. The TLGS-nutrition cohort is also notable for using a specifically designed and validated FFQ that was able to evaluate the usual dietary intakes of the Iranian population. Along with categorizing UPF consumption into tertiles, continuous variable analysis was conducted, which provides richer information and avoids biases [61]. Enrolled participants were free from CVD at baseline, which decreases the risk of diet modification in response to disease onset. As dietary patterns may change over time, the assessment of dietary intakes at one-time point may result in non-differential bias. Furthermore, it has been reported that considering only basal dietary data as an exposure yields weaker associations compared with cumulative averages [62]. To address this concern, analyzed data were gathered from the first time available nutritional data (3rd phase of TLGS) and further follow-ups at the fourth, fifth, and sixth phases up to March 2018. Finally, we selected the UPF based on the Nova food classification, a processing grade-based indicator of diet quality.

Of note, some points potentially limited our study. Although major-known confounding variables were adjusted in our models, there may still be residual or unmeasured confounders that may result in biased exposure effect estimates. As the Iranian food composition table was incomplete, we mostly used the USDA nutrient databank for the dietary analyses. Our validated FFQ has lacked data regarding some UPF i.e. instant noodles and energy bars. In addition, due to the differences in the food culture and dietary habits, it will be difficult to generalize our findings to other societies.

## Conclusion and policy implication

In conclusion, our study suggests a harmful association between UPF intake and CVD risk in a representative sample of Iranian adults. An increase of 50 g/day intake of UPF was accompanied by a 22% increased risk of CVD. Subgroup analysis suggested that ultra-processed salty snacks were directly associated with CVD. The adverse effect of UPF on CVD could be justified by considering specific nutritional aspects related to industrial processing conditions. The increasing tendency to consume UPF seems to be challenging in the coming years, therefore, encouraging to limit their consumption and replacing them with minimally processed foods by policy actions targeting industrial food processing approaches, marketing, and product reformulation would be appropriate strategies to reduce the burden of non-communicable diseases. Likewise, further studies are needed to assess the potential impact of food additives and neoformed compounds regarding these associations.

### Supplementary Information

**Additional file 1. Table S1**: Nova food classification: definition according to the extent and purpose of food processing.

**Additional file 2.** The list of FFQ items and selected UPF.

**Additional file 3. Table S3**: The list of Ultra-processed foods by subgroups according to the way of industrial production in Iran.

**Additional file 4. Table S4**: Baseline characteristics of the study participants based on CVD status (n=2050).
