# Supplementary material for: Higher ultra-processed food intake is associated with an increased incidence risk of cardiovascular disease: the Tehran lipid and glucose study
Source: Nutr Metab (Lond). 2024 Mar 19;21:14. doi: 10.1186/s12986-024-00788-x (PMC10949749; doi:10.1186/s12986-024-00788-x)
Supplement: Supplementary file 1 — Additional file 1. Table S1: Nova food classification: definition according to the extent and purpose of food processing. [file 12986_2024_788_MOESM1_ESM.docx]

Table S1. Nova food classification: definition according to the extent and purpose of food processing

| **Nova Food groups** | |
| --- | --- |
| **Unprocessed or minimally processed foods** | **Unprocessed:** Edible parts of plants (fruits, seeds, leaves, roots) or of animals (muscle, eggs, milk), and also fungi, algae and water, after separation from nature.  **Minimally processed:** unprocessed foods altered by industrial processes such as removal of inedible or unwanted parts, drying, crushing, grinding, fractioning, roasting, boiling, pasteurization, refrigeration, freezing, placing in containers, vacuum packaging, non-alcoholic fermentation, and other methods that do not add salt, sugar, oils or fats or other food substances to the original food. The main aim of these processes is to extend the life of unprocessed foods, enabling their storage for longer use, and, often, to make their preparation easier or more diverse. Infrequently, minimally processed foods contain additives that prolong product duration, protect original properties or prevent proliferation of microorganisms. |
| **Processed culinary ingredients** | Substances obtained directly from group 1 foods or from nature by industrial processes such as pressing, centrifuging, refining, extracting or mining. Their use is in the preparation, seasoning and cooking of group 1 foods. These products may contain additives that prolong product duration, protect original properties or prevent proliferation of microorganisms. |
| **Processed foods** | Products made by adding salt, oil, sugar or other group 2 ingredients to group 1 foods, using preservation methods such as canning and bottling, and, in the case of breads and cheeses, using non-alcoholic fermentation. Processes and ingredients here aim to increase the durability of group 1 foods and make them more enjoyable by modifying or enhancing their sensory qualities. These products may contain additives that prolong product duration, protect original properties or prevent proliferation of microorganisms. |
| **Ultra-processed foods** | Formulations of ingredients, mostly for industrial use only, resulting from a series of industrial processes, many of which require sophisticated equipment and technology. The processes that enable the production of ultra-processed foods include the fractionation of whole foods into substances, chemical modifications of these substances, the assembling of unmodified and modified food substances together using industrial techniques such as extrusion, molding and pre-frying, frequent application of additives, their function therein consists of making the final product palatable or hyper-palatable, and sophisticated packaging, usually with synthetic materials. Cosmetic additives such as flavors, flavor enhancers, colors, emulsifiers, sweeteners, thickeners and antifoams, fillers, carbonic acid, foaming, gelling and glazing agents; and additives that extend shelf life protect original properties or prevent the proliferation of microorganisms. Processes and ingredients used to create ultra-processed foods are designed to produce highly profitable products (low cost ingredients, long shelf life, emphatic branding), convenient (ready-to-eat), hyper-tasty snack products that can replace all other Nova food groups, especially group 1 foods. |

**Reference:**

Monteiro, C.A.; Cannon, G.; Levy, R.B.; Moubarac, J.-C.; Louzada, M.L.; Rauber, F.; Khandpur, N.; Cediel, G.; Neri, D.; Martinez-Steele, E.; et al. Ultra-processed foods: what they are and how to identify them. Public Health Nutr. 2019, 22, 936–941, doi:10.1017/S1368980018003762.
