# Supplementary material for: Higher ultra-processed food intake is associated with an increased incidence risk of cardiovascular disease: the Tehran lipid and glucose study
Source: Nutr Metab (Lond). 2024 Mar 19;21:14. doi: 10.1186/s12986-024-00788-x (PMC10949749; doi:10.1186/s12986-024-00788-x)
Supplement: Supplementary file 3 — Additional file 3. Table S3: The list of Ultra-processed foods by subgroups according to the way of industrial production in Iran. [file 12986_2024_788_MOESM3_ESM.docx]

**Table S3.** The list of Ultra-processed foods by subgroups according to the way of industrial production in Iran

| **Ultra-processed foods subgroups** | **Food items** |
| --- | --- |
| **Breads** | Baguette bread, Toast bread |
| **Salty snacks** | Crackers, Puff, chips |
| **Fast foods** | French fries, Hamburger, Kielbasa, Sausage, Pizza |
| **Sweetened beverages** | Industrial beverages, cola |
| **Sweets and desserts** | Cookies, Yazdi cake, Homemade cakes, other cakes, industrial jams, dried sweets, cream sweets, GAZ, Candies, SOHAN, Chocolate, Caramel cream, sesame pudding, NOGHL, Donuts |
| **Dairy** | Cacao milk, yogurt cream, cream cheese, traditional ice cream, non-traditional ice cream |
| **Others** | Margarine, Ketchup, Mayonnaise |
