# Supplementary material for: Higher ultra-processed food intake is associated with an increased incidence risk of cardiovascular disease: the Tehran lipid and glucose study
Source: Nutr Metab (Lond). 2024 Mar 19;21:14. doi: 10.1186/s12986-024-00788-x (PMC10949749; doi:10.1186/s12986-024-00788-x)
Supplement: Supplementary file 4 — Additional file 4. Table S4: Baseline characteristics of the study participants based on CVD status (n=2050). [file 12986_2024_788_MOESM4_ESM.docx]

**Table S4**. Baseline characteristics of the study participants based on CVD status (n=2050)

| **Baseline Characteristics** | **Total**  **(n = 2050)** | **CVD^+^**  **(n = 208)** | **CVD^-^**  **(n = 1842)** | ***P*** |
| --- | --- | --- | --- | --- |
| Age (year) | 46.28±11.34 | 57.24±10.15 | 45.04±10.79 | 0.001 |
| Male (%) ^#^ | 46.0 | 65.9 (137) | 43.8 (806) | 0.001 |
| BMI | 28.08±4.62 | 28.62±4.76 | 28.02±4.61 | 0.08 |
| WC (cm) | 92.99±11.98 | 98.27±11.24 | 92.39±11.91 | 0.001 |
| HTN (%) ^#^ | 16.9 | 38.6 (80) | 14.5 (264) | 0.001 |
| SBP | 114.77±17.72 | 127.71±20.82 | 113.30±16.71 | 0.001 |
| DBP | 75.27±10.81 | 79.93±12.84 | 74.74±10.43 | 0.001 |
| Serum cholesterol | 195.39±37.90 | 208.34±41.54 | 193.93±37.19 | 0.001 |
| Diabetes (%) ^#^ | 9.2 | 20.2 (42) | 7.9 (146) | 0.001 |
| Fasting serum glucose (mg/dL) | 94.97±25.95 | 106.73±37.61 | 93.64±23.94 | 0.001 |
| Current Smoking (%) ^#^ | 19.6 | 24.5 (51) | 19.0 (348) | 0.06 |
| Physical activity* (MET-hours/week) | 15.87 (4.24-39.69) | 13.89 (3.96-34.96) | 16.12 (4.27-40.12) | 0.53 |
| TG to HDL ratio | 4.23±3.30 | 4.92±3.01 | 4.15±3.33 | 0.001 |
| \| Data are mean ± SD unless stated otherwise (independent t-test and chi-square test were used for continuous and dichotomous variables, respectively. Given non-normal distributions, Mann-Whitney U test was applied).  * Data is median (IQR); ^#^ Data are shown in percent (%) and count numbers (n)  Footnotes: CVD (cardiovascular disease); BMI (body mass index); WC (waist circumference); HTN (hypertension); SBP (systolic blood pressure); DBP (diastolic blood pressure); FSG (fasting serum glucose); TG (triglyceride); HDL (high density lipoprotein lipase) \| \| --- \| | | | | |
